# P53-regulated long non-coding RNA TUG1 affects cell proliferation in human non-small cell lung cancer, partly through epigenetically regulating HOXB7 expression

**DOI:** 10.1038/cddis.2014.201

**Published:** 2014-05-22

**Authors:** E-b Zhang, D-d Yin, M Sun, R Kong, X-h Liu, L-h You, L Han, R Xia, K-m Wang, J-s Yang, W De, Y-q Shu, Z-x Wang

**Affiliations:** 1 Department of Biochemistry and Molecular Biology, Nanjing Medical University, Nanjing, Jiangsu, China; 2 Central Laboratory, Second Affiliated Hospital of Southeast University, Nanjing, Jiangsu, China; 3 Department of Oncology, First Affiliated Hospital of Nanjing Medical University, Nanjing, Jiangsu, China; 4 Department of Oncology, Second Affiliated Hospital of Nanjing Medical University, Nanjing, Jiangsu, China; 5 Department of Oncology, Affiliated Nanjing Hospital of Nanjing Medical Univeraity, Nanjing, Jiangsu, China

**Keywords:** p53, TUG1, proliferation, non-small cell lung cancer, HOXB7

## Abstract

Recently, a novel class of transcripts, long non-coding RNAs (lncRNAs), is being identified at a rapid pace. These RNAs have critical roles in diverse biological processes, including tumorigenesis. Here we report that taurine-upregulated gene 1 (*TUG1*), a 7.1-kb lncRNA, recruiting and binding to polycomb repressive complex 2 (PRC2), is generally downregulated in non-small cell lung carcinoma (NSCLC) tissues. In a cohort of 192 NSCLC patients, the lower expression of TUG1 was associated with a higher TNM stage and tumor size, as well as poorer overall survival (*P*<0.001). Univariate and multivariate analyses revealed that TUG1 expression serves as an independent predictor for overall survival (*P*<0.001). Further experiments revealed that TUG1 expression was induced by p53, and luciferase and chromatin immunoprecipitation (ChIP) assays confirmed that TUG1 was a direct transcriptional target of p53. TUG1 knockdown significantly promoted the proliferation *in vitro* and *in vivo*. Moreover, the lncRNA-mediated regulation of the expression of HOX genes in tumorigenesis and development has been recently receiving increased attention. Interestingly, inhibition of TUG1 could upregulate homeobox B7 (HOXB7) expression; ChIP assays demonstrated that the promoter of HOXB7 locus was bound by EZH2 (enhancer of zeste homolog 2), a key component of PRC2, and was H3K27 trimethylated. This TUG1-mediated growth regulation is in part due to specific modulation of HOXB7, thus participating in AKT and MAPK pathways. Together, these results suggest that p53-regulated TUG1 is a growth regulator, which acts in part through control of HOXB7. The p53/TUG1/PRC2/HOXB7 interaction might serve as targets for NSCLC diagnosis and therapy.

Lung cancer is the most frequent cause of cancer-related death worldwide. Non-small cell lung cancer (NSCLC) accounts for ∼85% of all cases and is generally diagnosed at an advanced stage.^1^ Lung carcinogenesis is a complicated biology process, which results from the dysregulation of many tumor-related genes.^2^ Therefore, a better understanding of the molecular mechanisms underlying NSCLC progression will supply an arm for improving the diagnosis and treatment of human NSCLC.

With the advent of next-generation sequencing technologies, it has become increasingly clear that long non-coding RNAs (lncRNAs) are pervasively transcribed in the genome.^3, 4^ LncRNAs represent a subgroup of non-coding RNAs that are >200 bases.^5^ Recent studies have shown that large numbers of lncRNAs are dynamically expressed in tissue-specific patterns, displaying weaker evolutionary constraint and lower expression levels than protein-coding genes.^6^ So far, a large range of lncRNAs are known to have important roles in cellular development, differentiation and many other biological processes.^7, 8, 9, 10, 11, 12^ Some lncRNAs have been found to be misregulated in various diseases, including Duchenne muscular dystrophy^10^ and heart diseases.^13^ The aberrant expressions of lncRNAs have also been shown in various types of cancer, including NSCLC.^14, 15, 16, 17^

The molecular mechanisms of lncRNAs are diverse. They could function (I) as decoys to locate transcription factors; (II) as regulatory signals for transcription; (III) as scaffolds to aggregate different proteins; (IV) as a ‘sponge' to interact with microRNAs; and (V) as guides to binding to chromatin-modifying enzymes to target genes.^10, 18^ Typical is the PRC2 (polycomb repressive complex 2).

Recently, numerous lncRNAs have been identified to have a direct role in recruiting PRC2. PRC2, a methyltransferase, which is composed of EZH2 (enhancer of zeste homolog 2), SUZ12 (suppressor of zeste 12) and embryonic ectoderm development, can catalyze the di- and trimethylation of lysine residue 27 of histone 3 (H3K27me3), thus modulating gene expression. These lncRNAs epigenetically regulate gene expression through binding to PRC2 in various biological processes, especially in cancer. For instance, HOTAIR, a 2.2-Kb lncRNAs involved in the repression of HOX loci, which promoted breast metastasis.^11, 14^ ANRIL, a 3.8-Kb lncRNA, is involved in silencing of p15^INK4B^.^19^ These show that many lncRNAs are associated with PRC2 complex, and that the dysregulation of PRC2-related lncRNAs participate in various pathological processes, including cancer.

Lately, Khalil *et al.*^20^ identified that ∼20% of the lncRNAs expressed in various cell types are bound to PRC2 by way of genome-wide RNA immunoprecipitation (RIP) analysis, including taurine upregulated gene 1 (*TUG1*). TUG1, a 7.1-kb lncRNA, was initially detected in a genomic screen for genes upregulated in response to taurine treatment of developing mouse retinal cells. The depletion of TUG1 in the developing mouse eye was found to block retinal development.^21^ These results indicate that TUG1 may be necessary for development, and that its dysregulation may participate in human disease progression. However, the biological functions of TUG1 in the control of NSCLC tumorigenesis have not been well characterized, which prompted us to explore the role of TUG1 in human NSCLC.

In the present study, we found that lncRNA TUG1 was significantly downregulated in NSCLC tissues compared with the corresponding non-tumor lung tissues and may serve as an independent predictor for the overall survival in NSCLC. In addition, TUG1 was a direct transcriptional target of p53 through interaction with the putative p53 response element in the promoter region of TUG1. Moreover, TUG1 could regulate cell growth both *in vitro* and *in vivo*. In addition, we demonstrated that TUG1 could epigenetically modulate homeobox B7 (HOXB7), which may partly account for TUG1-mediated proliferation regulation, thus affecting the proliferation of NSCLC both *in vitro* and *in vivo*.

## Results

### TUG1 expression is downregulated in human NSCLC tissues and correlates with poor prognosis

**Figure 1:**
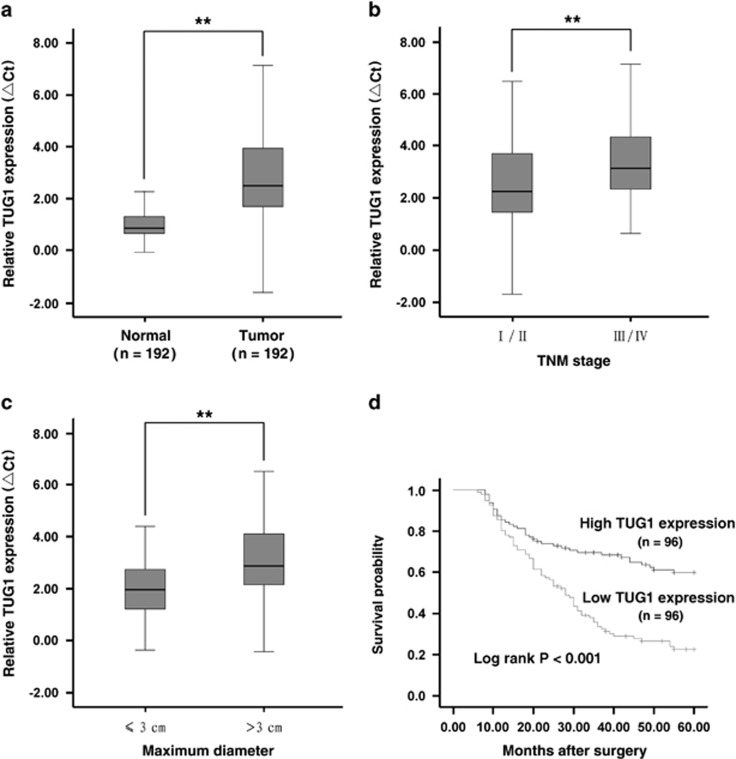
Analysis of TUG1 expression in NSCLC tissues and clinical parameters. (**a**) TUG1 was detected in 192 pairs of NSCLC tissues by qRT-PCR. The levels of TUG1 in NSCLC tissues are significantly lower than those in non-tumorous tissues. The ΔCt value was determined by subtracting the GAPDH Ct value from the TUG1 Ct value (relative to a single reference value). Smaller ΔCt value indicates higher expression. (**b** and **c**) Data are presented as relative expression level in tumor tissues (shown as ΔCt). TUG1 expression was significantly lower in patients with a higher pathological stage and big tumor size. (**d**) Patients with low levels of TUG1 expression showed reduced survival times compared with patients with high levels of TUG1 expression (*P*<0.001, log-rank test). **P<0.01

To detect the level of TUG1 expression, we analyzed TUG1 expression using qRT-PCR in 192 pairs of NSCLC tissues compared with the corresponding non-tumor tissues and found that TUG1 was significantly downregulated in 86% (166 of 192) of the cancerous tissues (downregulated by >50%, *P*<0.001) compared with normal counterparts, both in lung squamous cell carcinoma and lung adenocarcinoma tissues (Figure 1a). Next, we examined the correlation of TUG1 expression level with the clinical parameters in NSCLC. As shown in Figures 1b and c, the TUG1 downregulation was correlated with advanced pathological stage (*P*=0.002) and greater tumor size (*P*=0.003). Other clinical parameters, for example, sex (male and female), age (≤60 years and >60 years) and smoking status, were found not to be significantly correlated with TUG1 in our study (Supplementary Table S2).

Kaplan–Meier survival analysis and log-rank tests using patient postoperative survival were performed to further evaluate the correlation between TUG1 expression and the prognosis of NSCLC patients. The median ΔCt value for TUG1 in tumor tissues was used to divide the samples into high (below the median, *n*=96) and low (above the median, *n*=96) TUG1 expression group, and the corresponding *P*-value was calculated by log-rank analysis. From the Kaplan–Meier survival curve, we observed that patients with high levels of TUG1 expression had remarkably longer survival time than those with low levels (*P*<0.001, log-rank test; Figure 1d).

By univariate analysis, we identified three prognostic factors: histological grade (low, middle or high), TNM stage (I/II, III/IV) and TUG1 expression, whereas the other clinical parameters, such as lymph node metastasis (N0, N1 and above), age (≤60 years and >60 years), gender (male and female), tumor size (≤3 cm and >3 cm) and history of smoking were not significant prognosis factors. Furthermore, multivariate analysis revealed that the TUG1 expression could be regarded as a significant independent predictor of poor survival in NSCLC patients (*P*<0.001), as well as histological grade (*P*=0.002) and TNM stage (*P*<0.001) (Supplementary Table S3).

Taken together, these results suggested that downregulation of TUG1 may have important roles in NSCLC development and progression.

### TUG1 is induced by p53 and p53 interacts with the p53 response element in the promoter region of TUG1

**Figure 2:**
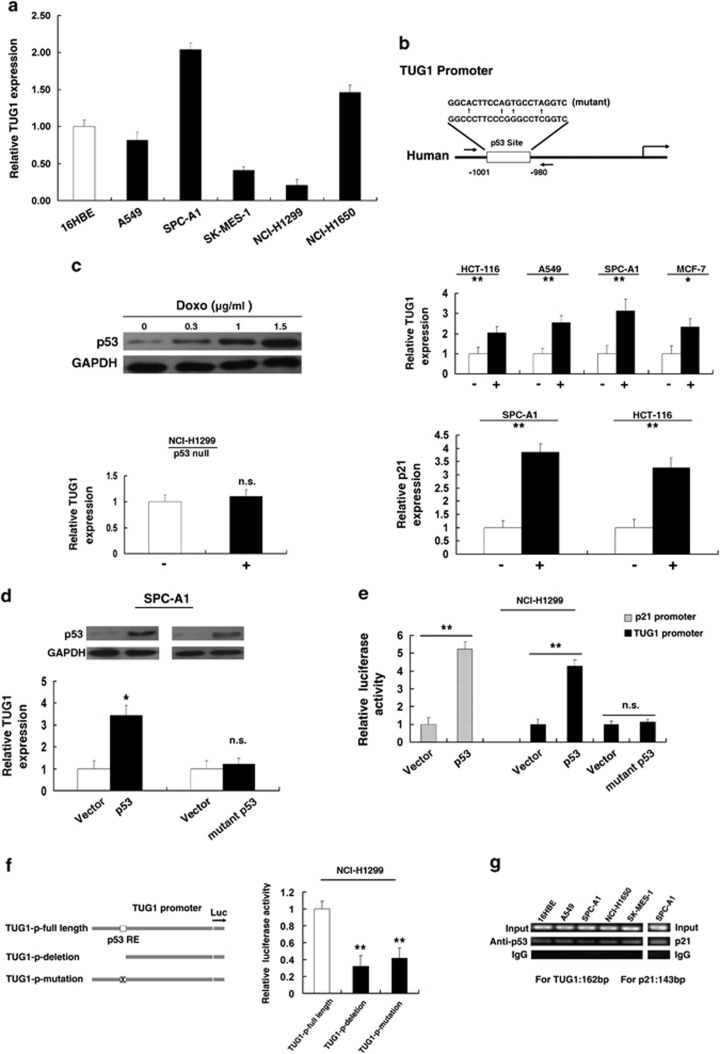
p53 induces TUG1 through interacting with the promoter region of TUG1. (**a**) Analysis of TUG1 expression levels in NSCLC cell lines (A549, SPC-A1, SK-MES-1, NCI-H1299 and NCI-H1650) compared with the normal bronchial epithelial cell line (16HBE) by qRT-PCR. (**b**) Description of p53RE and mutant p53RE in promoter region of TUG1. The position of ChIP primers (**e**) was indicated by arrows. (**c**) Western blotting was used to detect the p53 induction by doxo. qRT-PCR was used to detect the effect of doxo on TUG1 expression in p53-WT and p53-null cells. (**d**) Induction of TUG1 by ectopically expressed p53 (wild-type p53 or mutant p53). Overexpression was confirmed by western blotting. (**e**) Induction of TUG1 promoter activity by p53, but not mutant p53 in NCI-H1299 cell lines. (**f**) Deletion and mutation analysis of the promoter activity to determine the role of the p53RE in p53-mediated regulation of TUG1. (**g**) The p53 binding at the promoter regions of TUG1 was assessed by ChIP analysis. ChIP primers were detailed in Materials and Methods section. Shown are representative images of three independent experiments. Error bars indicate means±S.E.M. *P<0.05, **P<0.01. n.s., not significant

To explore the mechanism of low expression of TUG1, first, qRT-PCR was performed to detect the expression of TUG1 in diverse NSCLC cell lines. As shown in Figure 2a, three cell lines (A549, SK-MES-1 and NCI-H1299) expressed lower levels of TUG1 compared with the normal bronchial epithelial cell line (16HBE) and two cell lines (SPC-A1 and NCI-H1650) expressed relatively high endogenous levels of TUG1. Second, we analyzed the promoter region of TUG1 and detected the presence of the p53-binding sites (wild type (WT)), as shown in Figure 2b. We speculated that p53 could modulate TUG1 expression at the transcriptional level. Next, we treated HCT-116 cells expressing WT p53 (HCT-116 WT) with different concentrations of doxorubicin (doxo), a known DNA-damaging agent. After 24 h, western blot analysis was performed to detect the expression level of p53 and the results revealed that doxo induced p53 in a dose-dependent manner (Figure 2c). Next, we treated HCT-116 cells at 1.5 *μ*g/ml for 24 h and found that doxo could induce TUG1 expression. In addition, such induction was also detected in other cell lines expressing wild-type p53, that is, MCF-7, SPC-A1 and A549 (Figure 2c). To confirm the specific influence of p53 on TUG1 expression, we treated NCI-H1299 cells (a p53-null cell line) at the same concentration of doxo. Almost no induction was detected in p53-null cells (NCI-H1299) (Figure 2c). As doxo may induce cell response independent of p53, we enhanced p53 expression by transfecting a p53 expression vector (WT) and also found that enforced p53 expression increased the expression of TUG1 (Figure 2d), also in A549 cell line (Supplementary Figure S2A), similar to the induction of a noted p53-regulated gene, *p21*(Figure 2c). Next, we sought to determine whether p53 mutation could regulate TUG1 expression. Toward this end, p53 with a point mutation (R175H) at the DNA-binding domain, a frequent mutant in diverse cancer,^22^ had no impact on TUG1 expression, indicating that TUG1 is specifically induced by WT p53.

To determine whether p53 transcriptionally regulates TUG1, we cloned the promoter region (∼1.6 kb) of TUG1 into luciferase reporter plasmid (pGL3 basic). As shown in Figure 2e, the luciferase assays showed that p53 induced the promoter activity of TUG1, which was comparable with the induction of p21 promoter. In addition, we found that mutant p53 had no impact on the promoter activity of TUG1 (Figure 2e). To further determine the function of this p53RE, we made deletion at the promoter of TUG1 (Figure 2f). After transfection by p53 expression plasmid, the deletion (not containing the p53RE) caused significant reduction of promoter activity compared with the full-length promoter construct (Figure 2f). Furthermore, site-directed mutagenesis involving the conserved C and G of the p53RE (Figure 2b) also significantly reduced luciferase activity (Figure 2f).

To determine whether p53 can directly bind to the sites of TUG1 promoter *in vitro*, chromatin immunoprecipitation (ChIP) experiments were performed. As shown in Figure 2g, p53 immunoprecipitation was observed at the promoter of TUG1 in 16HBE, A549, SPC-A1, NCI-H1650 and SK-MES-1 cell lines. An isotype-matched IgG was used as a negative control, p21 served as a positive control for ChIP assay. The position of ChIP primers was indicated by arrows (Figure 2b).

Together, these results demonstrate that p53 interacts with the p53 response element in the TUG1 promoter, thus inducing its transcription.

### TUG1 regulates NSCLC cell proliferation both *in vitro* and *in vivo*

**Figure 3:**
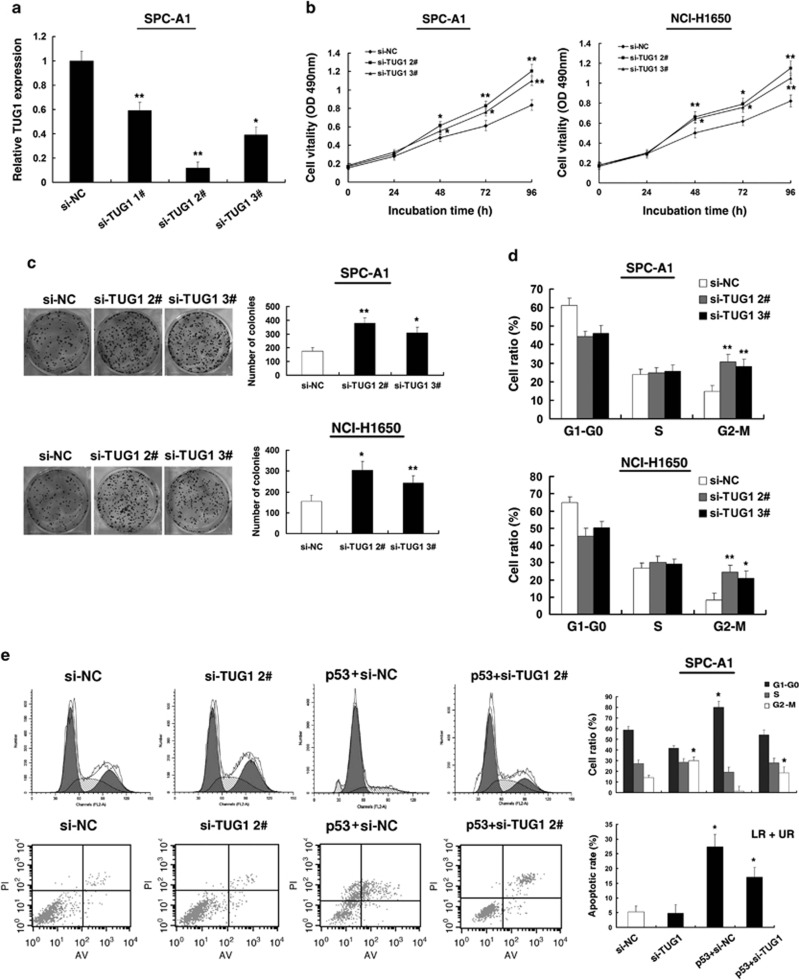
Effect of TUG1 on cell growth *in vitro.* (**a**) The relative expression level of TUG1 in SPC-A1 cells, transfected with si-NC or si-TUG1 (si-TUG1#1, #2 and #3), was tested by qPCR. (**b**) At 24 h after transfection, MTT assay was performed to determine the proliferation of SPC-A1 and NCI-H1650 cells. (**c**) Representative results of colony formation of SPC-A1 and NCI-H1650 cells transfected with si-NC or si-TUG1. (**d**) At 48 h after transfection, cell cycle was analyzed by flow cytometry. The bar chart represents the percentage of cells in G1–G0, S, or G2–M phase, as indicated. (**e**) SPC-A1 transfected with si-NC/si-TUG1/p53+si-NC and transfected with p53 followed by transfection with si-RNA TUG1. Forty-eight hours after transfection, cells were stained and analyzed by flow cytometry. LR, early apoptotic cells. UR, terminal apoptotic cells. Error bars indicate means±S.E.M. *P<0.05, **P<0.01

P53 is well known to induce many genes, such as p21, a noted tumor suppressor gene. To investigate the biological consequence of p53 induction of p53 of TUG1 in NSCLC cell, the relative high expression cell lines (SPC-A1, NCI-H1650) were selected for further study compared with the normal bronchial epithelial cell line (16HBE). First, TUG1 knockdown was performed using two different siRNAs (si-TUG1 2# and si-TUG1 3#) to avoid off-target effects (Figure 3a). Next, MTT assay showed that knockdown of TUG1 expression significantly increased cell proliferation both in SPC-A1 and NCI-H1650 cell lines compared with the control cells (Figure 3b). Next, colony-formation assay was performed to detect the cell viability. As shown in Figure 3c, the colony numbers of SPC-A1 and NCI-H1650 cells transfected with si-TUG1 were evidently higher than those transfected with si-NC. Flow cytometric analysis was performed to further examine whether the effect of TUG1 on proliferation of NSCLC cells by altering cell cycle progression. The results revealed that SPC-A1 and NCI-H1650 cells transfected with si-TUG1 could accelerate cell cycle progression (Figure 3d).

To further determine the physiological role of TUG1 in cells growth, SPC-A1 cells were transfected with p53 expression vector and followed by si-RNA TUG1 2#. After 48 h, cell cycle progression and apoptosis were analyzed by flow cytometric analysis. Our experiments showed that co-transfection of p53 and si-TUG1 could partly reverse p53-promoted growth arrest and apoptosis enhancement (Figure 3e). In addition, si-TUG1 2# showed a better suppressive effect. We thus chose to use si-TUG1 2# in the subsequent studies.

**Figure 4:**
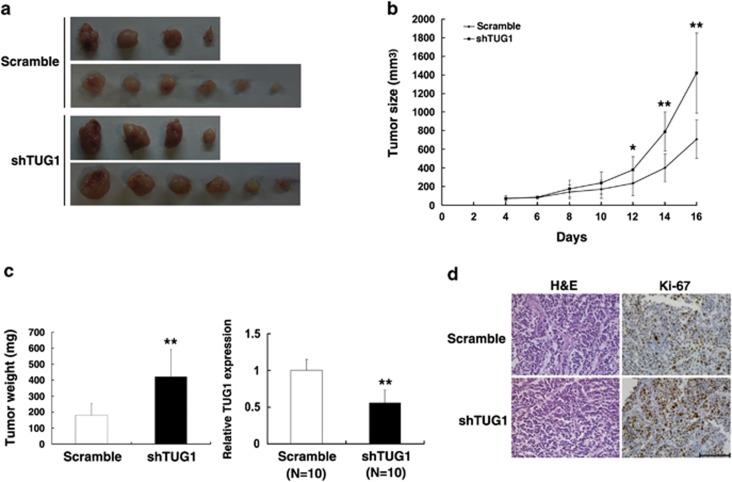
The impact of TUG1 on tumorigenesis *in vivo*. (**a** and **b**) Scramble or shTUG1 was transfected into SPC-A1 cells, which were injected in the nude mice (*n*=10), respectively. Tumor volumes were calculated after injection every 2 days. Bars indicate S.D. (**c**) Tumor weights are represented as means of tumor weights ±S.D. qRT-PCR was performed to detect the average expression of TUG1. (**d**) Histopathology of xenograft tumors. The tumor sections were under H&E staining and IHC staining using antibodies against Ki-67. Bar, 100 *μ*m. Error bars indicate means±S.E.M. *P<0.05, **P<0.01

To confirm whether the level of TUG1 expression affects tumorigenesis, scramble/shTUG1-transfected SPC-A1 cells were inoculated into nude mice. All of the mice developed xenograft tumors at the injection site. As shown in Figures 4a and b, tumor growth in the shTUG1 group was significantly more rapid than that in the control group. Up to 16 days after injection, the average tumor weight in the SPC-A1/shTUG1 group was markedly higher than in the control group. qRT-PCR analysis was conducted to detect the average expression of TUG1 in tumor tissues (Figure 4c). We also found that the tumors developed from SPC-A1/shTUG1 cells displayed higher Ki-67 staining than that in tumors formed by SPC-A1/scramble-transfected cells, as detected by immunohistochemistry (IHC) analysis (Figure 4d).

### TUG1 could participate in AKT and MAPK pathway by epigenetically regulating HOXB7

**Figure 5:**
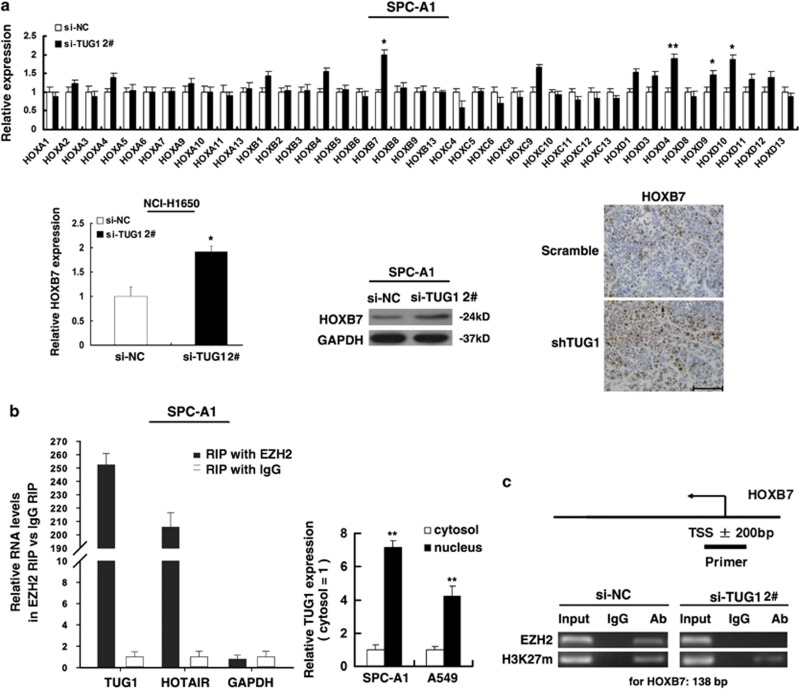
TUG1 could epigenetically regulate HOXB7 by binding to PRC2. (**a**) qRT-PCR was performed to detect the expression of HOX genes in transfected cells, and western blot assays were used to detect the level of HOXB7 after the transfection of si-TUG1. IHC assays were used to detect the HOXB7 in tumor sections from shTUG1-transfected cells. (**b**) RIP experiments were performed in SPC-A1 and the coprecipitated RNA was subjected to qRT-PCR for TUG1. HOTAIR was used as a positive control. The fold enrichment of TUG1 in EZH2 RIP is relative to its matching IgG control RIP. TUG1 nuclear localization, as identified using qRT-PCR in fractionated SPC-A1 and A549 cells. (**c**) ChIP of H3K27me3 and EZH2 of the promoter region of HOXB7 locus after siRNA treatment targeting si-NC or si-TUG1; qPCR were performed to determine the quantitation of ChIP assays. The levels of qPCR products are expressed as a percentage of the input DNA. Error bars indicate means±S.E.M. *P<0.05, **P<0.01

The importance of lncRNAs lies in regulating gene expression in human diseases. TUG1 may regulate genes expression through binding to PRC2. Recently, lncRNAs-mediated regulation on the expression of HOX gene family received increased attention in tumorigenesis and development.^11, 23, 24^ Many studies have shown that the HOX gene family were identified as classic modification targets of the polycomb complex during development, all four clusters were highly enriched in H3K27me3 marks.^25^ Moreover, large number of aberrant Hox gene expression have been found in various cancers.^26^ Next, we asked whether any of Hox genes were influenced by TUG1, thus participating in tumor progression. qRT-PCR analysis was performed to detect the expression of HOX genes. The results showed that HOXB7, HOXD4, HOXD9 and HOXD10 mRNA expression were induced in SPC-A1 cells transfected with si-TUG1 (Figure 5a). Next, we analyzed the expression of these genes using qRT-PCR in 50 pairs of NSCLC tissues (randomly selected according to each proportion of TNM stage in all the 192 patients) compared with the corresponding non-tumor tissues, and found that HOXB7 was upregulated in NSCLC tissues. No significant difference was found in the expression of HOXD4, HOXD9 and HOXD10 (Supplementary Figure S1). In addition, knockdown TUG1 also increased the expression of HOXB7 also in NCI-H1650 cell line. IHC analysis found that tumors developed from shTUG1 cells showed stronger HOXB7 staining than that in control (Figure 5a). To investigate the molecular mechanisms involved in the TUG1-mediated regulation of HOXB7, we examined the role of PRC2 in HOXB7 regulation. First, we validated that TUG1 could bind directly to PRC2 in SCP-A1 cells by RIP assay, as well as in SK-MES-1 cells (Supplementary Figure S2C); the co-precipitated RNA was subjected to qRT-PCR for TUG1 and HOTAIR served as a positive control (Figure 5b). In addition, we measured TUG1 expression in the nuclear and cytosolic fractions from SPC-A1 and A549 cell line s by qRT-PCR. The differential enrichments of GAPDH and U6 RNA were used as fractionation indicators (Supplementary Figure S2D). We found a considerable increase in TUG1 expression in the nucleus *versus* the cytosol (Figure 5b), thus suggesting that TUG1 is mainly localized in the nucleus and has a major regulatory function at the transcriptional level. The cells then transfect with an siRNA targeting the key catalytic subunit of PRC2 histone methyltransferase, EZH2, H3K27-trimethylated, which efficiently reduced the EZH2 level (Supplementary Figure S2B). The ChIP results show that primers directed at ±200 bp from the transcription start site of HOXB7 promoter detected H3K27 trimethylation and PRC2 binding in SPC-A1 cells transfected with si-NC. Knockdown TUG1 detected a loss of PRC2 binding and a decrease of H3K27 trimethylation occupancy (Figure 5c). These results suggest that TUG1 is required to target PRC2 occupancy and activity to regulate the transcription of HOXB7. These results suggested that TUG1 could epigenetically modulate the expression of HOXB7 by binding to PRC2.

**Figure 6:**
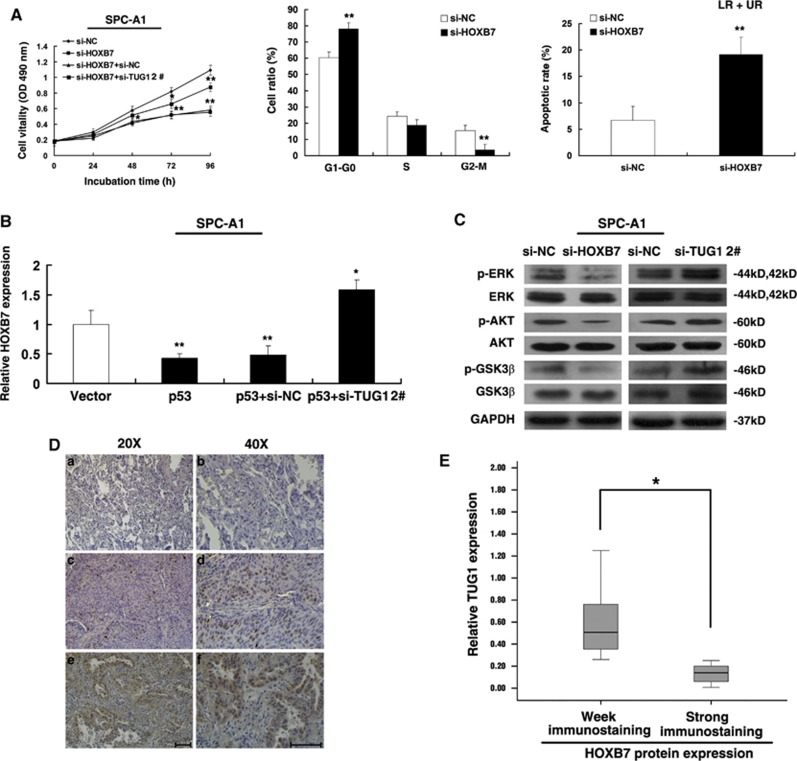
TUG1 could participate in AKT and MAPK pathway through modulating HOXB7. (**A**) MTT analysis of cell proliferation by co-transfection (si-NC, si-HOXB7, si-HOXB7+si-TUG1). At 48 h after transfection of si-HOXB7, the cell cycle and apoptosis were analyzed by flow cytometry. LR, early apoptotic cells. UR, terminal apoptotic cells. (**B**) qRT-PCR were performed to detect the expression of HOXB7 after overexpression of p53 and transfected with p53 followed by transfection with si-TUG1. (**C**) Western blotting analysis of the expression of p-ERK, total ERK, p-AKT, total AKT, p-GSK-3*β*, total GSK-3*β* proteins in indicated si-NC-transfected, si-HOXB7 and si-TUG1-transfected SPC-A1 cell lines. (**D**) Immunostaining of HOXB7 was negatively or very weakly positive in corresponding non-tumor lung tissues (a and b), but was strongly positive in squamous cell carcinoma tissues (c and d) and lung adenocarcinoma (e and f). Bar, 100 *μ*m. (**E**) The immunoreactivity of HOXB7 protein in NSCLC tissues showed a statistically significant inverse correlation with the relative level of TUG1 expression. Error bars indicate means±S.E.M. *P<0.05, **P<0.01

HOXB7, as a known oncogene,^27, 28, 29, 30^ has an important role in various types of cancer. To further probe the role of HOXB7 in NSCLC, we used siRNA to downregulate HOXB7 expression (Supplementary Figure S2B). MTT assays revealed that the cells transfected with si-HOXB7 had a significant growth inhibition compared with cells transfected with si-NC, and the co-transfection (si-TUG1 and si-HOXB7) could partially reverse si-HOXB7-induced growth inhibition (Figure 6A). Next, flow cytometric analysis indicated that the cell cycle progression of si-HOXB7 cells was stalled at the G1–G0 phases compared with cells transfected with si-NC and knockdown HOXB7 could induce apoptosis (Figure 6A). These findings are consistent with the effect of TUG1. Importantly, overexpression of p53 could inhibit HOXB7 expression and co-transfection (p53 and si-TUG1) could partially abrogate p53-induced HOXB7 inhibition (Figure 6B).

Furthermore, HOXB7 promoted cell proliferation through activating AKT and MAPK pathways.^28^ The western blotting revealed that the levels of p-ERK, p-AKT and p-GSK3*β* were decreased by knocking down of HOXB7 and were increased by knockdown of TUG1 in SPC-A1 cells, respectively (Figure 6C).

In addition, IHC was used to detect the expression of HOXB7 protien in NSCLC and corresponding non-tumor lung tissues. All of the tumors showed positive immunostaining of HOXB7 protein, both in lung squamous cell carcinoma and lung adenocarcinoma tissues: 12 of 50 NSCLC cases (24.0%) showed weakly positive staining and 38 NSCLC cases (76%) showed strongly positive staining. In contrast, all of the corresponding non-tumor lung tissues showed negative or weakly positive immunostaining of HOXB7 protein (Figure 6D). Further analysis revealed that the expression of TUG1 is inversely correlated with HOXB7 protein level in NSCLC tissues (Figure 6E).

These results suggest that TUG1 can participate in AKT and MAPK pathway through the modulation of HOXB7, by the binding to PRC2, indicating that TUG1 affects NSCLC cell growth at least partly through the epigenetic regulation of HOXB7.

## Discussion

It is becoming evident that mammalian genomes encode thousands of lncRNAs.^4^ In addition to microRNAs, lncRNAs are emerging as important factors in cell biology. To date, increasing evidence links dysregulation of lncRNAs to diverse human diseases including tumors.^31^

In our current study, we found that the average level of TUG1 in NSCLC tissues was significantly lower than those in corresponding non-tumor tissues. The low expression level of TUG1 in NSCLC patients was associated with advanced pathological stage and tumor size. Moreover, the low TUG1 expression in NSCLC tissues was associated with a poor prognosis and could be an independent prognostic indicator. However, TUG1 is overexpressed in bladder cancer, gastric cancer and osteosarcoma.^32, 33, 34^ This finding is probably because lncRNAs exhibit remarkably tissue-specific expression patterns than protein-coding genes.^6, 35^ These results indicate that TUG1 may have a tissue-specific expression pattern and exhibit important role in NSCLC development and progression.

The dysregulation of lncRNAs joins a wide variety of pathological processes, but the mechanisms of expression of lncRNAs are not clear and further exploration is required. Here, through bioinformation annlysis, we found that TUG1 promoter contained conserved p53-binding site, which was consistent with Khaki's predicted results.^20^ In addition, our results showed that TUG1 was a direct transcriptional target of p53. Our study indicated that the absence of p53 expression may contribute to the downregulation of TUG1 in NSCLC.

Although TUG1 has been studied in a variety of physiological and pathological processes, the possible role of TUG1 in NSCLC remains to be clarified. In our study, the function of TUG1 was investigated by RNA interference (RNAi)-mediated knockdown and p53-induced overexpression. As a result, inhibition of TUG1 could promote NSCLC cell proliferation both *in vitro* and *in vivo*. In addition, p53-mediated growth arrest and apoptosis induction was found to be partly reversed by exogenous RNAi TUG1. Our studies revealed that TUG1 may be a p53 downstream effector.

Many lncRNAs modulate specific gene loci through recruiting and binding to PRC2 protein complexes, and PRC2-mediated epigenetic regulation has a crucial role in the process of tumor development.^14^ Thus, TUG1 may elicit its biological activity through binding to PRC2, epigenetically regulating gene expression. Recently, lncRNAs-mediated regulation of the expression of HOX genes in tumorigenesis and development has received increasing attention. For example, HOTAIR interacts with PRC2 and is required for histone H3 lysine-27 trimethylation of HOXD locus. Wang *et al.*^23^ found that lncRNA HOTTIP binds to the adaptor protein WDR5 directly and targets WDR5/MLL complexes across HOXA, driving histone H3 lysine 4 trimethylation and activating gene transcription. As well-known modification targets of the polycomb complex due to their richness in H3K27me3 markers, HOX genes are essential for morphogenesis and development.^36^ The dysregulation of HOX gene expression has been shown in many diverse cancers.^26^ In addition, Ke *et al.*^37^ showed that H3K4me3 and H3K27me3 switches in HOX gene clusters between normal and prostate cancer cells through a genome-wide analysis of H3K4me3 and H3K27me3 modifications.

Inspired by the above fact, we detected the expression levels of the HOX gene family after TUG1 knockdown. Among the differentially expressed genes, we discovered that HOXB7 was upregulated in NSCLC tissues and had a closely related function in carcinogenesis. In addition, RIP assays confirmed that TUG1 could bind to PRC2 and ChIP assays validated that knockdown of TUG1 resulted in the loss of H3K27 trimethylation and PRC2 binding to the genomic loci of HOXB7, confirming that HOXB7 was a bona target of TUG1/PRC2-regulated genes.

In an attempt to understand the biological role of HOXB7 in NSCLC, we inhibited HOXB7 expression and found an apparent inhibition of proliferation. In addition, HOXB7 regulates cell growth mainly through the MAPK and PI3K/Akt activation pathways.^28^ TUG1 knockdown could also increase the levels of p-ERK, p-AKT and p-GSK3*β*. Moreover, our results showed that the expression levels of HOXB7 were upregulated in human NSCLC tissues and inversely correlated with the expression levels of TUG1. As a member of the HOX gene family, high levels of HOXB7 promote the proliferation rate in different types of tumors, highlighting the oncogenic feature of this gene.^28, 38, 39^ In addition, Rosenfeld and colleagues^40^ found that TUG1 with specific localization could relocate transcription units in a three-dimensional space of the nucleus in response to growth signals by binding to methylated Pc2 (Polycomb 2 protein), thus regulating the promoter activity of growth control gene. Our results showed a considerable increase in TUG1 expression in the nucleus compared with the cytosol (Figure 5b) and that TUG1 could epigenetically regulate HOXB7 (Figure 5c). In our study, the knockdown of TUG1 was found to result in anti-apoptotic activity; the lower expression of TUG1 strengthens this effect due to the loss of PRC2 binding and H3K27 trimethylation occupancy at the HOXB7 locus, similarly to the function of ncRNA intSMYD3.^41^ Moreover, epigenetic deregulation, especially histone modification, contributes to the deregulation of HOX genes in cancer.^26^ This negative correlation highlights the importance of TUG1, especially in NSCLC tumorigenesis. Our findings provide a novel potential mechanism through which HOXB7 boosts tumor cell proliferation.

The present study suggests that lncRNAs may also be a component of p53-regulatory network, similarly to protein-coding genes. For example, PANDA and lincRNA-p21, have been confirmed to be p53 transcription targets.^42, 43^ In addition, we demonstrated that the co-transfection (p53 and si-TUG1) could partially abrogate p53-mediated HOXB7 inhibition. Therefore, TUG1-mediated regulation of cell growth is at least in part through regulation of HOXB7. Collectively, we showed that TUG1 is an important prognostic factor for NSCLC patients and modulates NSCLC cell proliferation both *in vitro* and *in vivo* bioassays. TUG1 as a member of PRC2-mediated epigenetic regulation participates in the occurrence and development of NSCLC. Our study may supply a strategy for targeting with the p53/TUG1/PRC2/HOXB7 interaction as a novel therapeutic application for NSCLC patients.

## Materials and Methods

Cell culture, reagents, flow-cytometric analysis and expression plasmid were described in Supplementary Materials and Methods.

### RNA extraction and qRT-PCR analyses

The total RNA was extracted from tissues or cultured cells with TRIzol reagent (Invitrogen, Grand Island, NY, USA), according to the manufacturer's protocol. One microgram total RNA was reverse transcribed in a final volume of 20 *μ*l using random primers under standard conditions using PrimeScript RT Reagent Kit with gDNA Eraser (Takara, Dalian, China; RR047A). After the RT reaction, 1 *μ*l of the complementary DNA was used for subsequent qRT-PCR reactions (SYBR Premix Ex Taq, TaKaRa) according to the manufacturer's instructions. The results were normalized to the expression of GAPDH. The qRT-PCR and data collection were carried out on ABI 7500 real-time PCR system (Applied Biosystems, Foster City, CA, USA). The primer sequences are summarized in Supplementary Table S1.

### Subcellular fractionation location

The separation of the nuclear and cytosolic fractions was performed using the PARIS Kit (Life Technologies, Carlsbad, CA, USA) according to the manufacturer's instructions.

### Transfection of cell lines

NSCLC cell lines were transfected with specific siRNA oligonucleotides. siRNAs included: TUG1 siRNA 1# (sense 5′-GGGAUAUAGCCAGAGAACAAUUCUA-3′, antisense 5′-UAGAAUUGUUCUCUGGCUAUAUCCC-3′); siRNA 2# (sense 5′-GCUUGGCUUCUAUUCUGAAUCCUUU-3′, antisense 5′-AAAGGAUUCAGAAUAGAAGCCAAGC-3′); siRNA 3# (sense 5′-CAGCUGUUACCAUUCAACUUCUUAA-3′, antisense 5′-UUAAGAAGUUGAAUGGUAACAGCUG-3′); HOXB7 siRNA (sense 5′- CUAUUCGAUUUGAGUUUCCdTdT-3′, antisense 5′-GGAAACUCAAAUCGAAUAGdTdT-3′);^44^ and EZH2 siRNA (sense 5′-GAGGUUCAGACGAGCUGAUUU-3′, antisense 5′-AAAUCAGCUCGUCUGAACCUC-3′).^45^ Non-specific siRNA (si-NC) was purchased from Invitrogen. Typically, cells were seeded at six-well plates and then transfected the next day with specific siRNA (100 nM) and control siRNA (100 nM) by using Lipofectamine RNAi MAX, according to the manufacturer's protocol (Invitrogen). The shRNA sequence of TUG1 (5′-CAGCTGTTACCATTCAACTTCTTAA-3′) was cloned into pENTR/U6 vector.

### Cell proliferation assays

Cell proliferation was monitored using Cell Proliferation Reagent Kit I (MTT) (Roche, Basel, Switzerland). The transfected cells were plated in 96-well plates (3000 cells/well). Cell proliferation was determined every 24 h following the manufacturer's protocol. For the colony-formation assay, a certain number of transfected cells were placed into each well of a six-well plate and maintained in media containing 10% FBS for 2 weeks, replacing the medium every 4 days. Colonies were fixed with methanol and stained with 0.1% crystal violet (Sigma-Aldrich, St. Louis, MO, USA) in PBS for 15 min. The colony formation was determined by counting the number of stained colonies. Triplicate wells were measured in each treatment group.

### Xenograft study

SPC-A1 cells were transfected with shTUG1 or Scramble using Lipofectamine 2000 (Invitrogen). After 48 h, cells were collected and injected into either side of the posterior flank of the same female BALB/c nude mouse. The tumor volumes and weights were measured every 2 days in mice from the control (10 mice) or shTUG1 (10 mice) groups; the tumor volumes were measured as length × width^2^ × 0.5. Sixteen days after injection, the mice were killed and tumor weights were measured and used for further analysis. The TUG1 levels were determined by qRT-PCR.

### Western blot assay

The cells were lysed using mammalian protein extraction reagent RIPA (Beyotime, Haimen, China) supplemented with protease inhibitors cocktail (Roche) and PMSF (Roche). Fifty micrograms of the protein extractions were separated by 10% SDS-PAGE transferred to 0.22 mm nitrocellulose (NC) membranes (Sigma-Aldrich) and incubated with specific antibodies.The autoradiograms were quantified by densitometry (Quantity One software; Bio-Rad, Hercules, CA, USA). Anti-EZH2 was from Abcam (Hong Kong, China). Anti-phospho-AKT, anti-AKT, anti-phospho-ERK, anti-ERK, anti-phospho-GSK3b and anti-GSK3b were from Cell Signaling Technology (Boston, MA, USA). Anti-HOXB7 was from Abcam. Anti-p53 was from Santa Cruz Biotechnology (Dallas, TX, USA). Results were normalized to the expression of GAPDH (Rabbit anti-GAPDH).

### Immunohistochemistry

NSCLC tissues and non-tumor tissue samples were immunostained for HOXB7.^46^ Anti-Ki67 was from Santa Cruz Biotechnology.

### Luciferase reporter assay

The luciferase assays were performed using a luciferase assay kit (Promega, Madison, WI, USA) according to the manufacturer's protocol. Briefly, cells were first transfected with appropriate plasmids in 24-well plates. Next, the cells were collected and lysed for luciferase assay 48 h after transfection. The relative luciferase activity was normalized with renilla luciferase activity. The promoter region of TUG1 was PCR-amplified by TaKaRa LA Taq (Takara) with the primers TUG1-p-F (*Kpn*1 site) and TUG1-p-R (*Xho*1 site), and was subcloned into the pGL3 basic firefly luciferase reporter. The amplified PCR fragments were then used as a template for generating promoter constructs carrying deletions or mutation using respective primers. All PCR products were verified by DNA sequencing. All primers sequences are summarized in Supplementary Table S1.

### Chromatin immunoprecipitation assays

The ChIP assays were performed using EZ-CHIP KIT according to the manufacturer, s instruction (Millipore, Billerica, MA, USA). The EZH2 antibodies were obtained from Abcam. H3 trimethyl Lys 27 antibody was from Millipore. The ChIP primer sequences (forward) 5′-GCCCACATAAACACCTCACAAATGA-3′ and (reverse) 5′-CCTTGGAAGCCGGGAGTAGCT-3′ were used for amplify the promoter region of TUG1. The ChIP primer sequences (forward) 5′-CATCCCCACAGCAGAGGAGAA-3′ and (reverse) 5′-ACCCAGGCTTGGAGCAGCTA-3′ were used for p21. The ChIP primer sequences (forward) 5′-GTCCCTGCCTACAAATCATCC-3′ and (reverse) 5′-GGAAGCAAACGCACAAGAAGT-3′ were used for HOXB7.

### RNA immunoprecipitation

RIP experiments were performed using a Magna RIP RNA-Binding Protein Immunoprecipitation Kit (Millipore) according to the manufacturer's instructions. Antibody for RIP assays of EZH2 was from Abcam.

### Tissue collection and ethics statement

A total of 192 primary NSCLC tissues were collected from patients who had undergone surgery at the First Affiliated Hospital of Nanjing Medical University and the Second Affiliated Hospital of Nanjing Medical University between 2005 and 2007 (China). All patients did not receive chemotherapy or radiotherapy before surgery. The study was approved by the Ethics Committee of Nanjing Medical University, and it was performed in compliance with the Declaration of Helsinki Principles. Written informed consent was obtained for all patient samples. The animal experiments were performed with the approval of The Institutional Committee for Animal Research and in conformity with national guidelines for the care and use of laboratory animals.

### Statistical analysis

Student's *t*-test (two-tailed), one-way ANOVA analysis and Mann–Whitney test were performed to analyze the data using SPSS 17.0 software. Differences were considered to be statistically significant at *P*<0.05.

## Abbreviations

lncRNA: long non-coding RNA
TUG1: taurine-upregulated gene 1
PRC2: polycomb repressive complex 2
NSCLC: non-small cell lung carcinoma
EZH2: enhancer of zeste homolog 2
HOXB7: homeobox B7
ChIP: chromatin immunoprecipitation
RIP: RNA immunoprecipitation
