# Supplementary figures and images for: P53-regulated long non-coding RNA TUG1 affects cell proliferation in human non-small cell lung cancer, partly through epigenetically regulating HOXB7 expression
Source: Cell Death Dis. 2014 May 22;5(5):e1243–. doi: 10.1038/cddis.2014.201 (PMC4047917; doi:10.1038/cddis.2014.201)

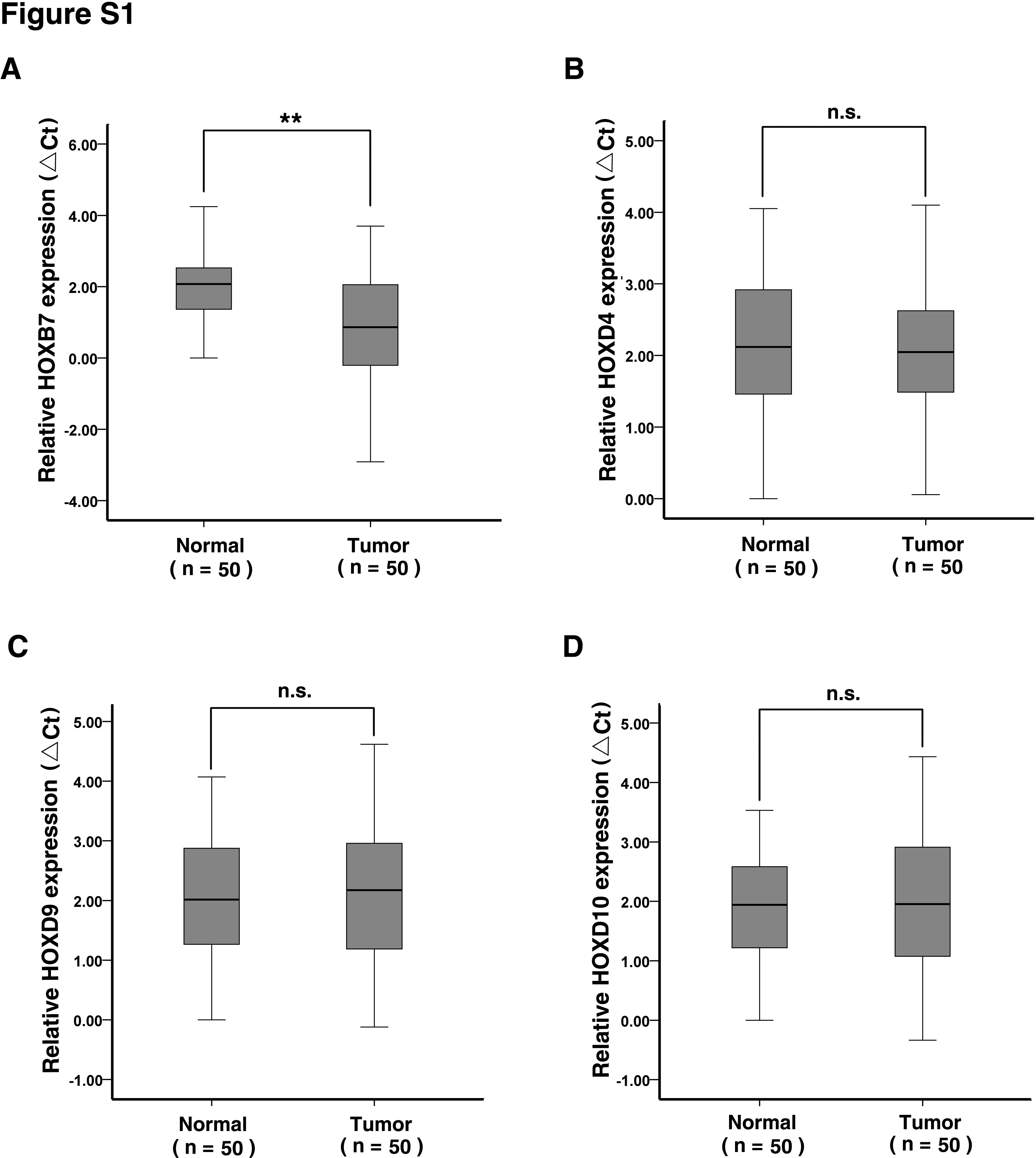

Supplement: Supplementary Figure S1 [file cddis2014201x1.tif]

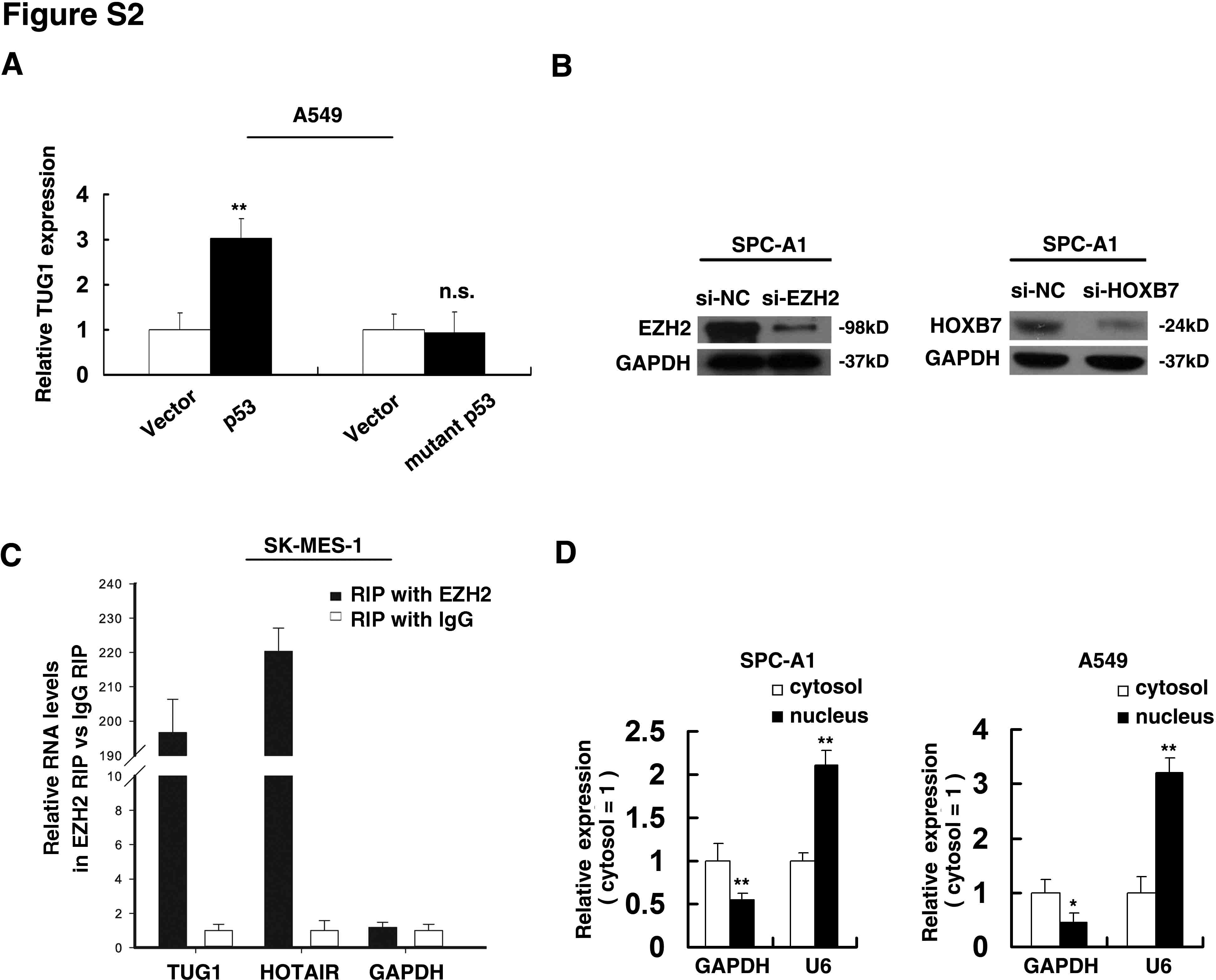

Supplement: Supplementary Figure S2 [file cddis2014201x2.tif]
