# Supplementary material for: P53-regulated long non-coding RNA TUG1 affects cell proliferation in human non-small cell lung cancer, partly through epigenetically regulating HOXB7 expression
Source: Cell Death Dis. 2014 May 22;5(5):e1243–. doi: 10.1038/cddis.2014.201 (PMC4047917; doi:10.1038/cddis.2014.201)
Supplement: Supplementary Materials and Methods [file cddis2014201x3.doc]

**Supplementary Materials and Methods**

**Cell culture**

Human NSCLC adenocarcinomas cell lines (A549, SPC-A1, NCI-H1650,NCI-H1299), a NSCLC squamous carcinomas cell line (SK-MES-1), and a normal human bronchial epithelial cell line (16HBE) were obtained from the Institute of Biochemistry and Cell Biology of the Chinese Academy of Sciences (Shanghai, China). Cells were cultured in RPMI 1640 or DMEM (GIBCO-BRL) medium supplemented with 10% FBS (Invitrogen), 100 U/ml penicillin, and 100 mg/ml streptomycin (invitrogen) in incubator at 37°C with 5% CO2.

**Reagents**

Doxorubicin hydrochloride (Doxo) was purchased from Sigma.

**Flow-cytometric analysis**

Transfected cells were harvested after transfection by trypsinization. After the double staining with fluorescein isothiocyanate (FITC)-Annexin V and propidium iodide was done by the FITC Annexin V Apoptosis Detection Kit (BD Biosciences) according to the manufacturer’s recommendations. The cells were analyzed with a flow cytometry (FACScan; BD Biosciences) equipped with a Cell Quest software (BD Biosciences). Cells were discriminated into viable cells, dead cells, early apoptotic cells, and apoptotic cells, and then the relative ratio of early apoptotic cells were compared with control transfection from each experiment. Cells for cell-cycle analysis were stained with propidium oxide by the CycleTEST PLUS DNA Reagent Kit (BD Biosciences) following the protocol and analyzed by FACScan. The percentage of the cells in G0–G1, S, and G2–M phase were counted and compared.

**Expression plasmid**

p53 (wild type) and mutant p53 (R175H) clone were purchased from Addgene.

**Supplementary Figure Legends**

**Figure S1 Expression of HOXB7, HOXD4, HOXD9 and HOXD10 in NSCLC tissues**

(A) The level of HOXB7 was upregulated in NSCLC tissues (n =50). Smaller ΔCt value indicates higher expression. (B) (C) and (D), the level of mRNA (HOXD4, HOXD9, HOXD10) in NSCLC tissues (n =50) was analyzed by qRT-PCR. n.s. not significant.

**Figure S2**

(A) Induction of TUG1 by ectopically expressed p53 (wild-type p53 or mutant p53) in A549 cell line. (B) Western blot assays detected the expression of EZH2 and HOXB7 after si-RNA transfection, respectively. (C) RIP experiments were performed in SK-MES-1 and the coprecipitated RNA was subjected to qRT-PCR for TUG1. HOTAIR was used as a positive control. The fold enrichment of TUG1 in EZH2 RIP is relative to its matching IgG control RIP. (D) After nuclear and cytosolic separation, RNA expression levels in SPC-A1 and A549 cells were measured by qRT-PCR. GAPDH was used as a cytosol marker and U6 was used as a nucleus marker. **, P < 0.01. n.s., not significant.
