# Supplementary material for: P53-regulated long non-coding RNA TUG1 affects cell proliferation in human non-small cell lung cancer, partly through epigenetically regulating HOXB7 expression
Source: Cell Death Dis. 2014 May 22;5(5):e1243–. doi: 10.1038/cddis.2014.201 (PMC4047917; doi:10.1038/cddis.2014.201)
Supplement: Supplementary Table S2 [file cddis2014201x5.doc]

| ***Supplementary Table S2****. TUG1 expression and clinical parameters* | | | |
| --- | --- | --- | --- |
| Factors | Tumor high expression  (N=96) N (%) | Tumor low expression  (N=96) N (%) | P |
| Age |  |  | 0.311^c^ |
| ≤60 years | 48（50.0%） | 41（42.7%） |  |
| ﹥60 years | 48（50.0%） | 55（57.3%） |  |
| Gender |  |  | 0.546^c^ |
| Male | 60（68.8%） | 64（60.4%） |  |
| Female | 36（31.2%） | 32（39.6%） |  |
| Smoking status |  |  | 0.460^c^ |
| Ever and current | 56（56.3%） | 61（65.6%） |  |
| Never | 40（43.7%） | 35（34.4%） |  |
| TNM stage |  |  |  |
| **Ⅰ** | 48（25.0%） | | |
| **Ⅱ** | 81（42.2%） | | |
| **Ⅲ** | 59（30.7%) | | |
| **Ⅳ** | 4（2.1%） | | |
| TNM stage |  |  | 0.001^c^ |
| **Ⅰ**and**Ⅱ** | 75（79.2%） | 54（55.2%） |  |
| **Ⅲ** and **Ⅳ** | 21（20.8%） | 42（44.8%） |  |
| Maximum diameter |  |  | <0.001^c^ |
| ≤ 3 cm | 43（40.6%） | 16（20.8%） |  |
| ＞3 cm | 53（59.4%） | 80（79.2%） |  |
| Histological grade |  |  | 0.043^c^ |
| Middle or High | 54（50%） | 40（47.9%） |  |
| Low | 42（50%） | 56（52.1%） |  |
| Histological classification |  |  | 0.104^c^ |
| SCC | 64（66.7%） | 53（55.2%） |  |
| AD or another | 32（33.3%） | 43（44.8%） |  |
| Lymph node metastasis |  |  | 0.042^c^ |
| Yes | 46(48.0%) | 60(62.4%) |  |
| No | 50(52.0%) | 36(37.6%) |  |

^a^ Age data are presented as the mean ± SD.

^b^ independent t-test.

^c^ Two-sided χ^2^ test.
