# Supplementary material for: P53-regulated long non-coding RNA TUG1 affects cell proliferation in human non-small cell lung cancer, partly through epigenetically regulating HOXB7 expression
Source: Cell Death Dis. 2014 May 22;5(5):e1243–. doi: 10.1038/cddis.2014.201 (PMC4047917; doi:10.1038/cddis.2014.201)
Supplement: Supplementary Table S3 [file cddis2014201x6.doc]

**Supplementary Table S3**

**Univariate and multivariate analysis of clinical parameters factors for overall survival in 192 patients with NSCLC**

| Risk factors | Univariate analysis | | | multivariate analysis | | |
| --- | --- | --- | --- | --- | --- | --- |
|  | HR* | p value | 95% CI | HR | p value | 95% CI |
| TUG1 expression | 1.322 | <0.001** | 1.189～1.469 | 1.286 | <0.001** | 1.147～1.442 |
| Histological grade (low, middle or high) | 2.016 | <0.001** | 1.372～2.963 | 1.855 | 0.002** | 1.259～2.733 |
| TNM stage (Ⅰ/Ⅱ, Ⅲ/Ⅳ) | 2.559 | <0.001** | 1.752～3.737 | 2.180 | <0.001** | 1.488～3.195 |
| lymph node metastasis (N0, N1 or above) | 1.429 | 0.069 | 0.973～2.099 |  |  |  |
| Tumor size (≤3 cm, >3 cm) | 1.296 | 0.231 | 0.847～1.983 |  |  |  |
| Histological classification (SCC, AD or another) | 1.371 | 0.103 | 0.938～2.004 |  |  |  |
| Age (≤60, >60) | 0.948 | 0.782 | 0.651～1.382 |  |  |  |
| History of smoking (ever, never) | 1.070 | 0.730 | 0.729～1.571 |  |  |  |
| Gender (male, female) | 0.902 | 0.612 | 0.607～1.342 |  |  |  |
| *HR hazard ratio |  |  |  |  |  |  |
| * p<0.05 |  |  |  |  |  |  |
| * * p<0.01 |  |  |  |  |  |  |
